# A foodborne outbreak of gastroenteritis caused by *Vibrio parahaemolyticus* associated with cross-contamination from squid in Korea

**DOI:** 10.4178/epih.e2018056

**Published:** 2018-11-13

**Authors:** Sun-Wha Jung

**Affiliations:** Environmental Health Division, Seoul Metropolitan Government, Seoul, Korea

**Keywords:** *Vibrio parahemolyticus*, Food, Retrospective studies, Prevalence, Korea

## Abstract

**OBJECTIVES:**

Water-borne diseases caused by *Vibrio parahemolyticus* are often known to cause gastritis when raw or undercooked seafood is eaten. It is very rare that* Vibrio *gastritis caused by ingesting non-seafood products occurs on a large scale. On September 19, 2017, a large-scale* Vibrio *gastritis occurred after the city residents consumed food at a bazaar held in a welfare center in Jungnang-gu, Seoul.

**METHODS:**

The total number of visitors was approximately 299, and 237 (79.3%) of them showed symptoms. Among those who showed symptoms, 116 (48.9%) consulted the hospital, and 53 (45.6%) were hospitalized. Among the 299 exposed individuals, 174 (58.1%) responded to this survey: 163 (93.6%) with and 11 (6.4%) without symptoms. This study was retrospectively conducted by investigating the exposed individuals. To investigate the spread of infection, medical staff of hospitals in the epidemic area were interviewed, exposed individuals surveyed, microbiological testing conducted, and ingredient handling and cooking processes investigated.

**RESULTS:**

A total of 237 individuals, including 6 food handlers, were affected (prevalence, 79.2%). During the microbiological testing, *V. parahemolyticus* was found in 34 patients and 4 food handlers. In the consumption analysis, the relative risk of kimbap was 6.79 (confidence interval 1.10 to 41.69). In-depth investigation found that squid, an ingredient of Korean pancake, and egg sheets, an ingredient of kimbap, were prepared using the same cutting board and knife, which were thought to be the cause of cross-contamination that led to a large-scale outbreak of* Vibrio *gastritis.

**CONCLUSIONS:**

A recent large-scale outbreak of* Vibrio *gastritis occurred due to the cross-contamination with kimbap during the preparation process of squid rather than the actual consumption of seafood. Thus, a more stringent hygiene management is necessary during the processing and management of food to prevent infections associated with *V. parahemolyticus*.

## INTRODUCTION

*Vibrio* is a genus of Gram-negative basophilic bacteria shaped like a banana or comma [1]. It is known as bacteria that exist mainly in the coastal regions or mouth of the rivers and has more than 100 types. Approximately 12 types cause infections in humans, including* Vibrio cholerae, Vibrio vulnificus, Vibrio parahaemolyticus*, and* Vibrio alginolyticus* [1,2]. Most modes of transmission are consuming undercooked seafood from the coastal regions or mouths of the rivers, which usually occur because of exposure to seawater [3,4]. *V. parahaemolyticus* is a seafood-mediated representative and a water-borne food-meditated bacterium, with >30 known O and K serotypes. Humans show characteristic hemolysin reaction, and 2 of these hemolysin genes promote diarrhea [1]. These genes are detected in >90% of patients but <1% in the environment or food [1]. They grow well in warm (>15℃) and low-sodium salt water (<25 parts per thousand sodium chloride) and proliferate very fast, with a doubling time of 8-9 minutes [2]. Its incubation period after food consumption is between 4 and 96 hours and is usually characterized by watery diarrhea, with nausea, abdominal pain, and vomiting [3,4]. The progression of illness lasts until 2-3 days, but can rarely lead to sepsis depending on the patient’s underlying illnesses or immune system [5-10]. The epidemic usually occurs during summer and late autumn when the temperature of the ocean surface increases.* Vibrio *gastritis is known to increase as the temperature increases [2,11,12]. The regional hospital reported to the Jungnang-gu health clinic because residents who consumed food in a bazaar held by a wellness center in Jungnang-gu, Seoul, on September 19, 2017, presented diarrhea, abdominal pain, and vomiting. After performing expedited tests of rectal smears on symptomatic persons that detected* Vibrio *bacteria, an epidemiological research with the health clinic was conducted. This study aimed to determine the source and mode of contamination of* Vibrio *gastritis in a bazaar serving raw seafood. This was not investigated during the epidemiological research.

## MATERIALS AND METHODS

### Research subjects

Twenty residents visited the emergency room of the community hospital because of diarrhea and stomach pain that occurred approximately at 7 p.m. on September 19, 2017. When the number of patients increased on the morning of September 20, 2017, the hospital reported the epidemic to the health clinic, which identified that this was a case of mass epidemic and that the common consumption among the symptomatic individuals within the region on September 19, 2017, was only the food provided between 11 a.m. and 5 p.m. at the bazaar. Thus, the residents who consumed food from the bazaar held by a welfare center in Jungnang-gu on September 19, 2017, were the identified subjects. For recruitment, we visited the regional hospital where the epidemic was reported on September 20, 2017. Epidemiological investigations, rectal smear tests, and medical staff interviews were conducted. Afterward, additional symptomatic patients were asked to report to the residents’ centers and health clinics.

### Research methods

#### Case definition

A case was defined as an individual who has more than 2 occurrences of diarrhea or vomiting among those who consumed food provided in the bazaar. Among the affected patients, confirmed cases were defined as suspected patients and symptomatic individuals with microbiology detected from the rectal smear.

#### Survey investigation

Consumption analysis was performed on 174 exposed individuals who responded to the epidemiological study conducted via surveys and phone interviews. This was a retrospective cohort study on exposed subjects. For statistical analysis, Microsoft Excel 2010 (Microsoft Corp., Redmond, WA, USA) and Epi Info 3.2 (Centers for Disease Control and Prevention, Atlanta, GA, USA) were used.

#### Microorganism test

An expedited test was performed on 5 symptomatic individuals in the epidemiological study conducted on September 20, 2017, and submitted to the Ministry of Food and Drug Safety. Moreover, a total of 60 human samples (54 symptomatic individuals and 6 food handlers) underwent rectal smear testing and tested positive for 10 types of bacteria (*Salmonella* spp., *Shigella* spp., pathogenic *Escherichia coli*,* Vibrio *spp., *Yersinia enterocolitica, Clostridium* spp., *Campylobacter* spp., *Bacillus cereus, Staphylococcus aureus*, and *Listeria monocytogenes*) and 5 types of virus (rotavirus, astrovirus, norovirus, sapovirus, and adenovirus) in the health clinic, and the results were submitted to the Seoul Research Institute of Public Health and Environment. A total of 36 environmental samples in 15 food samples (2 leftover pieces of kimbap, cooking water, and drinking water) and 21 cooking environmental samples (cooking tool knife, cutting board, and dish cloth) were all collected and requested for 10 types of bacterial tests in the Seoul Research Institute of Public Health and Environment. This study was conducted following the Korea Centers for Disease Control and Prevention research guidelines. Expedited test for *V. parahaemolyticus* was conducted using reverse transcription polymerase chain reaction. The process of testing for* Vibrio *was one-time alkaline peptone water enrichment at 37℃ for 6-8 hours, selective cultivation at 37℃ for 18-24 hours with thiosulfate-citrate-bile-sucrose agar-selective cultivation, and then pure cultivation at 37℃ for 18-24 hours in tryptic soy agar with 1%. API 20E, Vitek GNI+, Vitek II GN kit (bioMérieux, Marcy l’Etoile, France) were used for biochemical identification.

Squid and mackerel collected at the seafood shop were cultured for bacteria to identify the source of contamination. Pulsed-field gel electrophoresis (PFGE) was conducted on the cultured bacteria at the Seoul Metropolitan Government Research Institute of Public Health to examine the epidemiological relationship.

#### In-depth investigation of the cooking process

Food ingredients used at the bazaar were prepared by 6 people in 2 teams at a charcoal-fire barbeque restaurant on September 18, 2017, at the hall rather than the kitchen. For the in-depth investigation of the cooking process, 6 food handlers were investigated for ingredient handling and preparation process by assessing the cutting board and knife used at the charcoal-fire barbeque restaurant.

## RESULTS

### Scale of epidemic and clinical symptoms

The number of visitors in the bazaar was identified to be 299. A total of 237 visitors were affected: 102 local residents; 23 government workers from Myeonmok-dong units 3, 4, 7, and 8; 53 personnel related to the community corporation (Emergency Call Bank, Yurin); 20 workers at Myeonmok Social Welfare Center; 4 workers at Jungnang Elderly Welfare Center; 11 from the elderly social activity groups; 7 volunteers (including 6 food handlers); 3 from the welfare council; 5 civil society members in the Myeonmok-dong; 2 workers at the Green Hospital; 3 workers at Seoul North Municipal Hospital; and 4 workers at the community dining. Approximately 299 people visited the bazaar, and 237 cases were identified with a prevalence rate of 79.3%. Among the 237 patients, 116 (48.9%) had hospital consultation, and 53 (45.6%) of them were hospitalized. Furthermore, among the 299 exposed individuals, 174 (58.1%) responded to the epidemiological study: 163 symptomatic (93.6%) and 11 asymptomatic (6.4%). Among the 163 symptomatic patients, 125 were suspected and 38 were confirmed including the 4 food handlers. Symptoms included diarrhea (96.9%), abdominal pain (89.6%), fever (52.8%), nausea (62.6%), and vomiting (63.8%).

**Figure 1.**
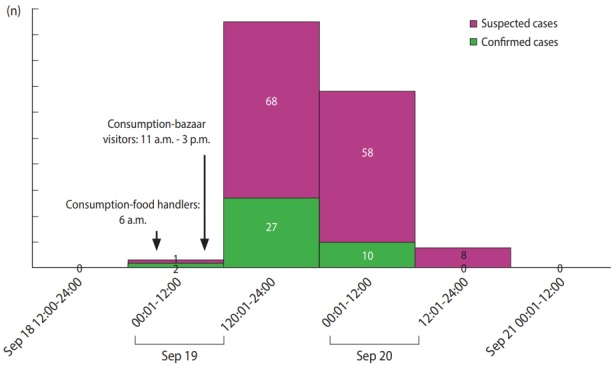
Epidemic curve about outbreak of enterocolitis caused by *Vibrio parahemolyticus*.

Three food handlers were the first subjects who became symptomatic. They made kimbap provided at the bazaar using the ingredients stored in the refrigerator at the restaurant hall at the dawn of September 19, 2017, and consumed the kimbap around 6 a.m. They did not consume any other food. Afterward, they visited the hospital due to severe diarrhea and abdominal pain, starting around 10 a.m. and could not participate in the bazaar. The bazaar convened from around 11 a.m. to 5 p.m. The epidemic started around 4 p.m. on the 19th and new patients continued to come to the hospital until around 12 p.m. on the 20th; the time with the highest number of new patients was between 7 p.m. on the 19th and 4 a.m. on the 20th (Figure 1). The incubation period between food consumption and symptom onset in food handlers, who were the first symptomatic persons, was 4 hours. The average incubation period of the epidemic after starting the bazaar was 7 hours. The minimum and maximum incubation periods were 4 and 36 hours, respectively.

#### Consumption analysis

Food provided at the bazaar was kimbap, feast noodles, fish cake soup, smoked chicken, Korean pancake, tteok boki, and rice cake. Among the 174 survey respondents, 163 (93.6%) were symptomatic and 11 asymptomatic (6.4%); thus, the consumption analysis was conducted (Table 1), which showed that the relative risk (RR) of kimbap was statistically significant at 6.79 (confidence interval [CI], 1.11 to 41.69), and that of other food items was not statistically significant.

Water supply was used for cooking and drinking, but was not statistically analyzed.

#### Microorganism test

For the microorganism test, rectal smear test was conducted on a total of 60 people including the food handlers. Among them, *V. parahaemolyticus* was detected in 34 symptomatic persons and 4 food handlers.

**Figure 2.**
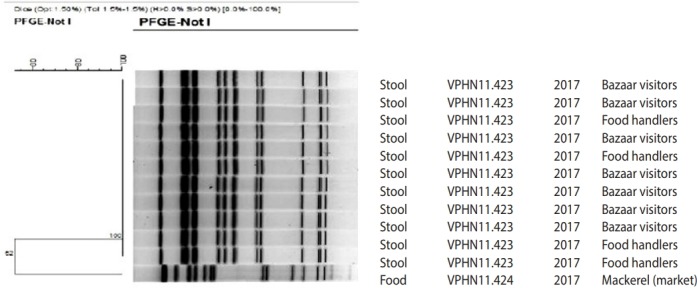
Pulsed-field gel electrophoresis (PFGE) for *Vibrio parahemolyticus* isolate from outbreak associated with cross-contamination from squid.

Bacteria were not detected in the collected kimbap and environment samples. Culture tests were performed on collected squids and mackerels from the same market, in which the squid was purchased in order to identify additional sources of contamination. The culture test did not find *V. parahaemolyticus* in the squid, but it was found in mackerel. PFGE analysis was performed on the bacteria detected in the mackerel and *V. parahaemolyticus* detected in the human samples in order to identify its relationship with *V. parahaemolyticus* that caused the epidemic. The results of PFGE analysis on 11 human samples and 1 mackerel sample on *V. parahaemolyticus* found no match between human samples and mackerel but found 2 types that were VPJN11.423 (11 samples) and VPJN11.424 (1 sample) (Figure 2).

#### In-depth investigation of the cooking process

Among the bazaar menu, smoked chicken and rice cake were provided via delivery on the 19th as finished products. Korean pancakes, fish cake soup, feast noodles, and tteok boki were prepared after starting the bazaar in a temporary cooking facility in the bazaar site. Ingredients for Korean pancakes and kimbap were prepared in 1 charcoal-fire barbeque restaurant on September 18, 2017, and kimbap was made on the dawn of September 19, 2017, and brought to the bazaar. Among the provided ingredients, squid was the only seafood item that was used in making Korean pancakes. The first patient had bacteria before starting the bazaar, and all 3 food handlers who ate the kimbap in the morning could not participate in the bazaar due to the onset of symptoms. For the other 3 food handlers who visited the bazaar, the symptoms started from 7 p.m. to 10 p.m.* Vibrio *was detected in 4 out of the 6 food handlers. Because the first patients had symptoms before the start of the bazaar and could not participate, we conducted an in-depth epidemiological research at the restaurant in which the ingredient preparation occurred the night before the bazaar to identify the possibility of cross-contamination of food items during the cooking preparation process. On the fourth day after the onset of the epidemic, the conditions of the hospitalized food handlers improved; therefore, an in-depth investigation of the cooking preparation process was conducted. The food was prepared in a charcoal-grilled barbeque restaurant, and bazaar food ingredients were prepared on a table at a hall rather than at the kitchen. Six food handlers prepared the ingredients using cutting boards and knives by dividing into 2 teams, consisting of 3 handlers each. According to the cooking process investigation, cutting of various vegetables and ingredients for kimbap was performed in the morning of the 18th. Around 3 p.m., defrosted and gutted squids were delivered; therefore, they were cut using 1 knife and cutting board that were used to cut the ingredients for the kimbap. Then, the used knife and cutting board were washed with water, and the sheet of eggs that were fried previously in the kitchen were cut using the same knife and cutting board. The squid that was cut as the ingredient for the Korean pancakes was blanched, and the strips of egg sheets for the kimbap were stored at room temperature until around 6 p.m., and then they were placed in the refrigerator.

## DISCUSSION

The large-scale epidemic in a bazaar held in a wellness center in Jungnang-gu is thought to have occurred due to the contamination of kimbap during the cooking preparation process. The basis for this assumption are as follows. First, the consumption analysis shows that the RR of kimbap was statistically significant at 6.79 (95% CI, 1.11 to 41.69). Second, the 3 food handlers, who were the first symptomatic persons, showed symptoms approximately 10 a.m. before the start of the bazaar after eating only kimbap in the dawn of the same day, and* Vibrio *was detected in 2 food handlers. The symptomatic food handlers could not participate in the bazaar that started around 11 a.m. due to the hospital visit. The epidemic is thought to be due to food rather than food handler contamination. Third, according to an in-depth epidemiological research on the cooking process, the egg sheets for kimbap and squids were prepared using the same knife and cutting board.

The duration between the food handlers consumed kimbap and the onset of symptoms in residents after food consumption was between 4 and 36 hours, which matches the previously known incubation period for* Vibrio *gastritis, i.e., between 4 and 96 hours. This short incubation period is thought to be because the proliferation of* Vibrio *bacteria had already occurred in the kimbap.

*V. parahaemolyticus* can be found in seawater, plankton, deposits in the ocean coast, coastal areas, and approximately 30 types of seafood, such as clams, oysters, shrimp, crabs, and squids [17,18].

In Korea, 10 cases of* Vibrio *gastritis have been reported in 2017, which occurred on a small scale with >90% due to consumption of seafood in a regular restaurant [19]. Most cases of* Vibrio *gastritis occurred due to the consumption of seafood, and a large-scale onset due to cross-contamination has not been reported in Korea. Although bacterial tests were conducted on the contamination of instant food items and sandwiches, cases of* Vibrio *have not yet been identified [20,21]. Overseas, the possibility of epidemic cross-contamination due to *V. parahaemolyticus* that occurred after eating cooked seafood has been reported [22]. Results that quantify the simulation on the cross-contamination of *V. parahaemolyticus* that occurs due to inappropriate hygiene management and behavior reported an average prevalence rate of 3.5% for the probability of *V. parahaemolyticus* cross-contamination occurring in the process of cooking ark shell in the kitchen, and for the primary contamination due to contact rather than food, cutting board is known to cause the highest cross-contamination among hands, knives, and cutting board [23]. Bacteria were not detected in the kimbap collected from the bazaar, because the test was performed on the remaining 2 pieces of kimbap and the bacteria were not detected. In this epidemic, the possibility of cross-contamination due to using the same knife and cutting board was inferred. However, an in-depth investigation of the cooking process was conducted rather than a reenactment; therefore, the possibility of cross-contamination through the hands of food handlers cannot be completely excluded.* Vibrio *bacteria have a very fast proliferation speed at room temperature; thus, seafood has to be refrigerated under 5℃ [16]. The doubling time of *V. parahaemolyticus* is 8-9 minutes; hence, in the case of cross-contaminated egg sheets, storing them at room temperature of 21-27℃, the average temperature in September, for 2-3 hours before refrigerating can amplify the proliferation of bacteria from even a small amount of contamination [2]. In the case of Korean pancakes that used squid as the ingredient, the possibility of* Vibrio *bacteria contamination, which is susceptible to heat, was decreased. The number of* Vibrio *bacteria is known to decrease to an undetectable level if heated for 10 minutes in a 50℃ water [24]. Other types of *V. parahaemolyticus* were detected in a different mackerel collected to determine the source of contamination, and PFGE analysis results confirmed 2 types: VPJN11.423 (11 samples) and VPJN11. 424 (1 sample). According to the results of the PulseNetKorea DB, these 2 types were firstly found in Korea.* Vibrio *bacteria are known to increase in concentration with increased temperature of seawater, thus increasing seafood contamination [25]. During summer and early autumn when the temperature of the seawater increases, many types of* Vibrio *can survive. The detection rate of* Vibrio *in non-sealed and non-frozen seafood is higher than that in sealed seafood, and such items should be displayed separately in the market to prevent cross-contamination among them [26]. Afterward, food handlers were educated about the sterilization and separation of knives and cutting boards, which resulted in no more patients having the infection within the region. One of the limitations of this investigation is that this the number of people who visited the bazaar was not clearly identified due to a sudden and large-scale epidemic, which made the identification of the exact number of exposed persons and their characteristics difficult due to out-of-contact. Whether the drinking water was consumed was not analyzed. We also restructured the epidemic with an in-depth investigation through interviews rather than a perfect reenactment; therefore, investigation on the contamination through the cook’s hands was inadequate. Despite this, the epidemic investigation was restructured through an epidemiological statistical analysis, on-site interview, and in-depth investigation close to a reenactment to identify the path and process of how a large-scale epidemic of* Vibrio *gastritis is possible from consuming kimbap that was cross-contaminated by the squid. *V. parahaemolyticus* can also bring a large-scale epidemic from cross-contamination without actually consuming the contaminated seafood. Furthermore, *V. parahaemolyticus* can induce cross-contamination from the market to the cooking process, and thus a more stringent hygiene management is needed.

## Figures and Tables

**Table 1.** Attack rates and RRs of gastroenteritis among 174 persons having meals^1^

Meal	Exposed			Non-exposed			RR (95% CI)
	No. of cases	Total	Attack rate (%)	No. of cases	Total	Attack rate (%)	
Kimbap	162	167	97.0	1	7	14.2	6.79 (1.11, 41.69)
Feast noodles	105	109	96.3	58	65	89.2	1.07 (0.98, 1.18)
Fish cake soup	64	66	96.9	97	108	89.8	1.08 (1.00, 1.17)
Smoked chicken	48	51	94.1	115	123	93.5	1.01 (0.93, 1.09)
Korean pancake	116	123	94.3	47	51	92.1	1.02 (0.93, 1.12)
Tteok boki	60	67	89.5	103	107	96.2	0.93 (0.85, 1.01)
Rice cake	29	34	85.2	134	140	95.7	0.89 (0.77, 1.02)

RR, relative risk; CI, confidence interval.

1 Date on Sep 19, 2017.

## SUPPLEMENTARY MATERIAL

Supplementary Material: Korean version is available at http://www.e-epih.org/.
