# Supplementary material for: A foodborne outbreak of gastroenteritis caused by Vibrio parahaemolyticus associated with cross-contamination from squid in Korea
Source: Epidemiol Health. 2018 Nov 13;40:e2018056. doi: 10.4178/epih.e2018056 (PMC6367201; doi:10.4178/epih.e2018056)
Supplement: Supplementary file 1 [file epih-40-e2018056-supplementary.pdf]

Volume: 40, Article ID: e2018056, 6 pages
https://doi.org/10.4178/epih.e2018056

A foodborne outbreak of gastroenteritis caused by *Vibrio parahaemolyticus* associated with cross-contamination from squid in republic korea

초록

배경: *Vibrio parahemolyticus* 에 의한 수인성 매개 질환의 경우 흔히 해산물을 날 것으로 먹거나 덜 익힌 경우 위장관염이 발생하는 것으로 알려져 있다. 비해산물의 섭취로 인한 비브리오 장염이 대규모로 유행이 발생하는 것은 매우 드물다. 2017년 9월 19일에 서울시 중랑구의 한 복지관에서 개최한 바자회에서 지역 주민들이 음식을 섭취한 후 대규모 비브리오 장염이 발생하였다.

연구 방법 및 대상: 바자회에 참석한 인원은 299명으로 추정되며 유증상자는 237명(79.3%)였다. 유증상자 중 병원진료를 받은 사람은 116명(48.9%)으로 이 중 입원환자는 53명(45.6%)이었다. 노출자 299명 중 174명(58.1%)이 설문에 응하였으며 이 중 유증상자 163명 (93.6%), 무증상자가 11명(6.4%)였다. 연구 디자인은 노출자 조사를 통한 후향적 코호트로 하였다. 유행조사 방법으로는 유행 발생 지역내 병원의 의료진 면담과 노출자에 대한 설문조사, 미생물검사, 재료 준비 및 조리 과정을 심층 조사하였다.

결과: 바자회에 참석한 노출자는 299명으로 추정되며 이 중 환례는 조리종사자 6명을 포함하여 237명으로 발병률은 79.2%였다. 시행한 미생물 검사상 환례 34명, 조리종사자 4명에서 *Vibrio parahemolyticus* 가 검출되었다. 섭취력 분석상 김밥이 상대 위험비(RR) 6.79(CI 1.10-

Volume: 40, Article ID: e2018056, 6 pages
https://doi.org/10.4178/epih.e2018056

41.692)였다. 심층 조사 결과 부침개의 재료인 오징어와 김밥 재료인 계란 지단을 같은 도마와 칼을 사용하였고 그 과정에서 교차오염 발생으로 비브리오 장염의 대규모 유행이 생긴 것으로 생각한다.

결론: 이번 비브리오 장염 대규모 유행은 해산물의 섭취가 아닌 오징어를 손질하는 과정에서 김밥에 교차 오염이 생겨 발생한 것으로 *Vibrio parahemolyticus* 의 경우 음식물을 만드는 과정과 관리에 보다 철저한 위생관리가 필요하다.

중심단어 : 비브리오 장염, 감염경로, 교차감염

서론

Volume: 40, Article ID: e2018056, 6 pages
https://doi.org/10.4178/epih.e2018056

Vibrio 속은 그람 음성 세균이며 바나나형 또는 콤마형 모양의 세균으로 호염기성 세균이다 [1]. 해안이나 강하구에 많이 존재하는 세균으로 알려져 있으며 100개가 넘는 종을 가지고 있다. 대략 12개의 종이 인간에게 감염을 일으키고 그 중 대표적인 것으로는 *V. cholerae, V. vulnificus, V. parahemolyticus, V. alginolyticus* 가 있다[1-2]. 감염경로는 흔히 연안이나 강하구의 해산물을 날 것으로 섭취하거나 덜 익혀 먹는 경우 또는 바닷물에 노출되어 생기는 경우가 대부분이다[3-4]. *V. parahemolyticus* 의 경우 해산물을 매개로 하는 수인성 식품 매개 질환의 대표적인 균이다. 혈청형은 O와 K serotype로 구분되며 30개 이상이 알려져 있다. 사람의 경우 특징적인 hemolysin reaction을 보이며 설사 질환에 있어 2개의 hemolysin gene이 관여한다[1]. 환자의 90% 이상에서 이 genes이 검출되나 환경이나 음식에서는 1% 미만에서 분리된다[1]. 따뜻하고(>15°c.) 저농도의 소금물(<25 ppt NaCl)에서 잘 자라고 증식 속도가 매우 빨라 복제시간이 8-9분으로 알려져 있다[2]. 음식 섭취 후 병의 발생의 잠복기는 4-96시간으로 수양성 설사를 주소로 하며 메스꺼움, 복통이나 구역 등의 증상이 생길 수 있다[3-4]. 병의 경과는 2-3일 이후 호전되나 드물게는 환자의 기저질환이나 면역상태에 따라 패혈증이 발생하는 경우도 있다[5-10]. 유행 시기는 해수면의 온도가 상승하는 여름과 늦가을에 주로 유행한다. 기온의 상승이 증가하면 비브리오 장염이 증가하는 것으로 알려져 있다[2,11-12]. 2017년 9월 19일에 서울시의 중랑구에서 한 복지관이 주최한 바자회에서 음식을 섭취한 주민들이 설사, 복통, 구토 증상을 보여 지역 병원에서 중랑구 보건소로 신고를 하였다. 유증상자 직장도말 신속 검사상 비브리오균 검출 결과를 듣고 보건소와 함께 역학조사를 시행하였다. 역

학조사를 통하여 날것의 해산물이 제공되지 않은 바자회에서 발생한 비브리오 장염에 대한 감염원과 감염경로를 찾고자 하였다.

연구 대상 및 방법

1. 연구 대상

9월 19일 당일 저녁 7시경부터 지역 사회 병원에 설사와 복통 증세로 지역주민 20여명이 응급실을 방문하였고 9월 20일 오전에도 환자가 늘자 병원에서 보건소에 유행 발생을 신고하였다. 보건소에서는 집단 유행 발생으로 판단하였고 9월19일 전후로 지역내 유증상자의 공통 섭취력은 9월 19일 오전 11시부터 오후 5시까지 진행된 바자회에서 제공한 음식외에는 없었음을 확인하였다. 이에 이번 유행의 연구대상은 2017년 9월19일 중랑구의 한 복지관에서 주최한 바자회의 음식을 섭취한 지역주민으로 하였다. 대상의 수집방법으로는 유행이 신고된 9월20일에 지역 병원을 방문하여 역학조사 및 유증상자 직장 도말 검사를 하였고 의료진 면담을 하였다. 이후 추가 유증상자는 9월23일까지 보건소와 각 동주민센터에 신고하도록 하였다.

2. 연구방법

1) 환례 정의

환례는 2017년 9월 19일 복지관에서 주최한 바자회에서 제공한 음식을 섭취한 자 중 하루 2회 이상의 설사 또는 구토 증상이 있는 자로 정의하였다. 환례 중 증상만 있는 경우를 의사 환자로 정하였고 증상과 함께 직장도말 검사상 미생물이 검출된 자를 확진 환자로 하였다.

2) 설문조사

섭취력 분석은 노출자중 역학조사에 응한 174명을 대상으로 하였으며 조사 방법은 설문지와

전화 인터뷰로 이루어졌다. 연구 디자인은 노출자 조사를 통한 후향적 코호트 조사를 하였으며 통계 방법으로 Microsoft Excel과 epi info를 사용하였다.

3) 미생물 검사

9월20일 시행한 역학조사에서 유증상자 5명에 대한 신속검사를 시행하여 식약처에 의뢰하였다. 또한 보건소에서 인체 검체 총60명(유증상자 54명, 조리 종상자 6명)을 직장도말로 세균10종(세균 10종: Salmonella spp., Shigella spp.,Pathogenic E.coli, Vibrio spp., Yersinia enterocolitica, Clostridium spp., Campylobacter spp., Bacillus cereus, Staphylococcus aureus, Listeria monocytogenes)과 바이러스5종(로타 바이러스, 아스트로바이러스, 노로바이러스 , 사포바이러스, 아데노바이러스) 검사를 시행하여 서울시 보건 환경 연구원에 의뢰하였다. 환경 검체는 총36건으로 남은 김밥 2조각과 조리수와 음용수 포함하여 식품 검체 15건과 조리 환경 검체 21건으로 (조리도구 칼, 도마, 행주 등)을 모두 수거하여 세균 10종 검사를 서울시 보건 환경 연구원에 의뢰하였다. 실험은 질병관리본부 실험 통합지침에 의거하여 수행하였으며 *V. parahemolyticus* 신속검사는 RT-PCR로 시행하였고 비브리오균의 검사과정은 37˚c 에서 6-8시간 1배 alkaline peptone water증균하여 선택배양을 37˚c 에서 18-24시간동안 TCBS 선택배양 후 순수배양은 37˚c 에서 18-24시간하며 1% NaCl 함유된 tryptic soy agr에 배양을 하였다. 생화학적 동정은 API 20E,Vitek GNI+,Vitek II GN Kit를 사용하였다.

감염원을 찾고자 해산물가게에서 수거한 오징어와 고등어는 세균 배양검사를 하였고 배양된 균은 역학적 연관성을 확인하기 위하여 간헐영역겔전기이동(Pulsed-field gel electrophoresis, PFGE) 을 서울시보건환경연구원에서 시행하였다.

4) 조리과정 심층 조사

바자회에서 사용할 음식 재료의 준비는 9월 18일날 숯불 고기집을 하는 식당에서 이루어졌으며 식당의 부엌이 아닌 홀에서 6명이 두 팀으로 나뉘어 재료준비를 하였다. 조리과정 심층 조사는 조리종사자 6명을 대상으로 숯불 고기집 식당에서 당시 사용했던 도마와 칼을 놓고 재료 손질 및 준비 과정을 심층 조사 하였다.

결과

1) 발생규모 및 임상증상

바자회에 참석한 대상은 총 299명으로 파악되었고 그 중 환례는 지역주민 102명, 면목동 3,4.7,8동 공무원 23명, 지역사회 회사(ECB,유린 원광)관련 53명, 면목사회복지관 직원20명, 중랑노인복지관 직원 4명, 노인 사회 활동 소속 11명, 자원 봉사자 7명(조리종사자6명포함), 복지 협의체 소속 3명, 면목4동 사회단체 소속 5명, 녹색 병원 직원 2명, 북부병원 직원 3명, 지역사회 식당 직원 4명으로 총 237명였다. 바자회에 참석한 인원은 299명으로 추정되며 환례는 237명으로 발병률은 79.3%였다. 환례 237명에서 병원진료를 받은 사람은 116명(48.9%)으로 이 중 입원환자는 53명(45.6%)이었다. 또한 노출자 299명중 174명(58.1%)이 역학조사에 응하였으며 이 중 환례는 163명(93.6%)이었고 환례가 아닌 경우는 11명(6.4%)이었다. 환례 163명 중 의사 환자는 125명였고 확진 환자는 조리종사자 4명을 포함하여 38명이었다. 증상으로는 설사(96.9%), 복통 (89.6%), 발열감 (52.8%), 메스꺼움 (62.6%), 구토 (63.8%) 등이 있었다.

최초 환자는 3명의 조리종사자로 바자회에 제공할 김밥을 만들기 위해 9월 19일 새벽에 식당

홀에서 냉장고에 보관된 김밥재료로 김밥을 만들었고 오전 6시경에 만든 김밥을 섭취하였고 그 외에는 섭취한 음식은 없었다. 이후 오전 10시경부터 심한 설사와 복통이 생겨 병원 진료를 받았으며 바자회는 참석하지 못하였다. 바자회는 11시경부터 5시경까지 진행되었다. 유행은 19일 오후 4시경부터 발생하여 20일 오후 12시경까지 환자 발생이 지속되었으며 가장 많은 환자가 발생한 시간은 19일 오후7시에서 20일 새벽 4시경이었다 (figure1). 최초 증상자인 조리 종사자의 음식섭취 후 증상발현까지의 잠복기는 4시간이었고 바자회 시작 후 집단 유행의 평균 잠복기는 7시간이었다. 최소 잠복기는 4시간, 최대 잠복기는 36시간이었다.

2) 섭취력분석

바자회에서 제공한 음식은 김밥, 잔치국수, 어묵탕, 훈제치킨, 부침개, 떡뽁이, 절편이었다. 설문에 응한 자는 174명이었으며 환례는 163명(93.6%), 환례가 아닌 경우는 11명(6.4%)였고 이들을 대상으로 섭취력 분석을 하였다.(Table.1) 섭취력 분석상 김밥이 상대위험비(RR)가 6.791(CI 1.106-41.69)로 통계적으로 유의하였으며 다른 음식은 통계적으로 유의하지 않았다.

조리수와 음용수는 상수도를 사용하였고 음용수에 대한 통계분석은 시행하지 못하였다.

3) 미생물검사

미생물검사는 조리종사자 포함하여 총 60명을 직장 도말 검사하였고 그 중 유증상자 34건과 조리종사자 4건에서 Vibrio parahemolyticus 가 검출되었다.

수거한 김밥과 환경 검체에서는 균은 검출되지 않았다. 추가 감염원을 찾고자 오징어를 구입한 가게에서 다른 오징어와 고등어를 수거하여 배양검사를 하였다. 배양검사 결과 오징어에서는 나오지 않았으나 고등어에서 Vibrio parahemolyticus 가 검출되었다. 집단 유행을 일으킨

Vibrio parahemolyticus 와 연관성을 알기위해 고등어에서 검출된 균과 인체검체에서 나온 Vibrio parahemolyticus 에 대하여 PFGE 분석을 하였다. Vibrio parahemolyticus에 대한 인체검체 11주와 고등어 1주를 PFGE분석 결과 인체검체와 고등어는 일치하지 않았고 VPJN11.423(11주)와 VPJN11.424(1주) 2개의 유형이 확인 되었다. (Fig2)

3) 조리과정 심층조사

바자회 식단 중 훈제치킨, 절편은 완제품으로 19일 당일 배달되어 공급되었고 부침개,어묵탕과 잔치국수, 떡복이는 바자회장에 마련된 임시조리시설에서 바자회 시작 후 만들어서 제공되었다. 부침개 재료와 김밥은 9월18일 숯불 갈비집을 하는 한 식당에서 재료준비를 하였고 9월 19일 새벽에 김밥이 만들어져 바자회에 가져갔다. 공급된 재료 중 해산물은 오징어가 있었고 오징어를 사용한 메뉴는 부침개가 있었다. 최초의 환자가 바자회 시작 전에 발생하였고 오전에 김밥을 먹은 조리종사자 3명 모두 바자회 시작 전에 증상 발생으로 바자회에 참석하지 못하였다. 바자회에 참석한 다른 종사자 3명의 경우 모두 오후 7시-10시경에 증상이 시작되었다. 조리종사자 6명중 4명에서 비브리오가 검출 되었다. 최초의 환자가 바자회 시작 전에 생기고 바자회에 참석하지 못하였기에 조리준비과정에서 음식물의 교차오염의 가능성을 확인하고자 바자회 전날 조리준비를 했던 식당에서 심층역학조사를 하였다. 유행 발생 4일째 입원했던 조리종사자들의 상태가 호전되어 조리 준비 과정을 심층조사 하였다. 조리 준비 장소는 기존 숯불고기집을 운영하는 식당으로 식당 내 부엌이 아닌 홀의 식탁에서 바자회 음식재료를 준비하였다. 6명의 조리 종사자가 3명씩 나누어 2개의 도마와 칼을 이용하여 음식물 재료를 준비하였다. 조리과정 조사상 18일 오전에는 바자회에 쓸 각종 채소 다듬기와 김밥 속재료 썰기를 하

Volume: 40, Article ID: e2018056, 6 pages
https://doi.org/10.4178/epih.e2018056

였다. 오후 3시경에 내장이 손질된 해동된 오징어가 배달되어 김밥 속재료를 썰었던 1개의 칼과 도마를 이용하여 오징어를 썰었다. 이후 이용한 칼과 도마를 물로 씻고 부엌에서 부쳐놓았던 계란 지단을 같은 칼과 도마 위에 놓고 지단을 썰었다. 부침개의 재료로 썰어놓았던 오징어는 끓는 물에 데치고 김밥 속재료와 김밥에 쓰일 계란 지단은 상온에 보관하다가 오후 6시경에 이후에 냉장고에 보관하였다.

고찰

이번 중랑구의 복지관에서 주최한 바자회의 비브리오 대규모 유행은 조리 준비 과정에서 생긴 교차감염으로 김밥이 오염되어 생긴 것으로 생각한다. 그 근거로는 첫째 섭취력 분석에서 김밥이 상대위험비(RR)가 6.791(CI 1.106-41.69)로 통계적으로 유의하였다.둘째 최초의 증상자 조리종사자 3명이 당일 새벽 김밥만을 먹고 바자회 시작 전인 10시경에 증상을 보였으며 조리종사자 2명에서 비브리오 균이 검출되었다. 증상이 발생한 조리종사자의 경우 병원 진료로 11시경부터 시작한 바자회에 참석하지 않았으며 조리 종사자에 의한 오염보다 오염된 음식에 의한 유행으로 생각한다. 셋째 조리과정에 대한 심층 역학 조사상 김밥에 쓰인 계란지단과 오징어가 같은 도마와 칼을 사용하였다.

조리종사자의 김밥 섭취시간과 지역주민이 바자회에서 음식 섭취 후 증상 발현까지의 시간은 4시간에서 36시간으로 기존의 비브리오 장염의 잠복기인 4시간에서 96시간과 일치하였으나 비교적 잠복기가 짧게 발생한 것은 섭취한 당시 이미 김밥내에 상당수의 비브리오균이 증식되어 있었기 때문이라 생각한다.

Volume: 40, Article ID: e2018056, 6 pages
https://doi.org/10.4178/epih.e2018056

*Vibrio parahemolyticus*는 바닷물, 플랑크톤, 바다연안의 침적물과 조개,굴, 새우, 게, 오징어 등 30여개의 해산물에서 발견된다[17,18].

우리나라의 경우 비브리오 장염은 2017년 10건의 발생이 보고 되었으며 90%이상이 일반음식점에서 해산물을 섭취하여 소규모로 발생하였다[19]. 비브리오 장염의 경우 대부분 해산물을 섭취하여 발생하였으며 교차감염에 의한 대규모 발생은 국내에 보고된 바가 없었으며 즉석식품이나 샌드위치에 대한 교차감염에 대한 연구로 균 검사를 하였으나 비브리오가 검출된 보고는 없었다 [20,21]. 외국의 경우 익힌 해산물을 먹고 생긴 *Vibrio parahemolyticus* 에 의한 유행의 교차감염의 가능성을 대하여 제기한 바 있다[22]. 부적절한 위생관리와 행위로 인해 생기는 *Vibrio parahemolyticus* 의 교차 감염에 대한 모의실험을 정량화한 결과 부엌에서의 피조개의 조리 과정에서 *Vibrio parahemolyticus* 교차 감염이 생길 발생률을 평균 3.5%로 보고하였고 음식이 아닌 접촉으로 인한 1차 교차감염의 경우 손, 칼,도마 중 도마가 가장 높은 교차감염을 일으키는 것으로 알려져 있다 [23]. 바자회에서 수거한 김밥의 경우 균은 검출되지 않았는데 이는 잔존식으로 남은 김밥 2조각을 검사한 것으로 균이 검출되기 어려웠을 것으로 생각한다. 이번 유행의 경우는 같은 칼과 도마를 사용하여 교차감염의 가능성을 추정하였으나 조리 과정 심층조사에서는 재연이 아니라 과정에 대한 심층 조사로 조리 종사자의 손에 의한 교차감염의 가능성을 완전히 배제하지 못하였다. 비브리오균은 상온에서 증식속도가 매우 빨라 해산물의 경우 5°c 미만에서 냉장 보관하도록 되어 있다[16]. *Vibrio parahemolyticus* 의 복제시간은 8-9분으로 교차오염 된 계란지단의 경우 적은 양의 오염이라도 9월 평균온도인 21°c-27°c 상온에서 2-3시간 보관하다가 냉장 보관한 것은 균의 증식을 증폭시켜 계란지단의

오염을 더욱 증가시켰을 것으로 생각한다[2]. 오징어를 재료로 사용하였던 부침개의 경우 오징어를 데쳐서 사용하였기 때문에 열에 약한 비브리오가 부침개를 통해서 감염될 가능성은 떨어졌다. 비브리오의 경우 50°c 물에 10분간 가열되면 검출되지 않은 정도로 균의 수가 감소되는 것으로 알려져 있다[24]. 감염원을 찾고자 수거한 다른 고등어에서 다른 균주의 *Vibrio parahemolyticus*가 검출되었으며 PFGE 분석결과 확인된 VPJN11.423(11주)와 VPJN11.424(1주) 2개의 유형으로 이 두개의 유행은 PulseNetKorea DB 확인결과 국내에서 처음으로 확인된 유형이었다. 비브리오균의 경우 바닷물의 온도가 높아질수록 균의 밀도가 높아지는 것으로 알려져 있고 이에 해산물의 오염도 증가한다[25]. 바닷물의 온도가 올라가는 여름과 초가을의 경우 여러 균주의 비브리오가 생존할 수 있으며 포장된 해산물의 경우보다 비포장 된 얼리거나 냉장상태의 해산물에서 비브리오의 검출률을 높은 것으로 알려져 있고 각 해산물간의 교차오염을 방지하기 위하여 해산물의 판매 단계부터 분리되어 전시할 필요성이 있다고 하였다[26]. 이후 조리 종사자에게 칼, 도마 소독과 분리 사용에 대한 위생교육을 하였으며 더 이상의 지역내 환자는 발생하지 않았다. 조사의 제한점으로는 갑작스런 대규모의 유행으로 바자회의 참석한 불특정 다수를 대상으로 시행한 조사로 연락두절과 연락처 미확인의 어려움으로 정확한 노출자수와 인적 사항를 파악하기가 어려웠다. 또한 음용수 섭취여부에 대한 통계분석을 시행하지 못하였으며 보다 완벽한 재연보다 심층면담조사로 유행을 재구성하여 조리종사자의 손에 의한 오염에 대한 조사가 미흡하였다. 그럼에도 불구하고 역학적 통계분석 및 현장 인터뷰 그리고 재연에 가까운 심층조사를 통하여 유행을 재구성하였으며 이를 통하여 오징어로 인한 교차 오염된 김밥의 섭취로 비브리오 장염의 발생이 어떻게 대규모의 유행이 생길 수 있는지에

Volume: 40, Article ID: e2018056, 6 pages
https://doi.org/10.4178/epih.e2018056

대한 경로와 과정을 알아내고자 하였다. *Vibrio parahemolyticus* 의 경우 오염된 해산물을 섭취하지 않고 교차오염으로도 대규모의 유행을 가지고 올 수 있다. 또한 *Vibrio parahemolyticus* 의 경우 판매부터 조리과정까지 모든 단계에서 해산물의 교차 감염을 일으킬 수 있어 보다 철저한 위생관리가 필요하리라 생각된다.

참고문헌

1. David L. Heymann.Control of communicable disease manual. 20th edition p102-p111

2. Baker-Austin, Trinanes , Gonzalez-Escalona , Martinez-Urtaza. Non-cholera Vibrios: The Microbial Barometer of climate change. Trends Microbiol. 2017 Jan; 25(1):76-84

3. Su YC, Liu C .Vibrio parahaemolyticus: a concern of seafood safety. Food Microbiol. 2007 Sep; 24(6):549-58.

4. Letchumanan, Chan, Lee Vibrio parahemolyticus: A review on the pathogenesis, prevalence, and advance molecular identification techniques. Front Microbiol. 2014 Dec 11; 5: 705

5. Rajeev R Fernado, Sujatha Krishnan, Morgan G Fair weather and Charles Ericsson Vibrio parahemolyticus septicaemia in a liver transplant patient: a case report. Fernado et al. Journal of Medical Case Reports 2011,5:171

6. Liu Y, Tam YH, Yuan J, Chen F, Cai W, Liu J,et a A Foodborne Outbreak of Gastroenteritis Caused by Vibrio parahaemolyticus and Norovirus through Non-Seafood Vehicle: PloS

one,2015:e0137848

7. Hwang HY, Jeong SH, Lee SU, Jeong TJ, Choi BG, Kim MH.A Case of Vibrio parahaemolyticus Septicemia in a Patient with Liver Cirrhosis. Korean J Clin Microbiol. 2000 Mar; 3(1):79-81.

8. Son JS, Park IG, Uh Y, Jang IH, Yoon KJ, Baik SK.A case of Bacteremia Caused by Vibrio parahaemolyticus in Liver Cirrhosis. J Clin Pathol Qual Control. 1998 Dec; 20(2):383-387. Korean.

9. Park IG, Uh Y, Jang IH, Yoon KJ, Jang WI, Lee JI.A Case of Bacteremia Caused by Vibrio parahaemolyticus. Korean J Clin Pathol. 1997 Apr; 17(2):303-307. Korean.

10. Kim JS, Park SY, Kil YC, Lee HJ, Suh JT. A Case of Simultaneous Isolation of Vibrio parahaemolyticus and Vibrio alginolyticus. Korean J Clin Microbiol. 2000 Sep; 3(2):147-152. Korean.

11. McLaughlin JB, DePaola A, Bopp CA, Martinek KA, Napolilli NP, Allison CG, et al. Outbreak of Vibrio parahaemolyticus Gastroenteritis Associated with Alaskan Oysters: NEJM 2005; 353:1463-1470

12. Yoon KS, Min KJ, Jung YJ, Kwon KY, Lee JK, Oh SW .A model of the effect of temperature on the growth of pathogenic and nonpathogenic vibrio parahemolyticus isolated from oysters in korea. Food Microbiology 25(2008)635-41

13. Cheon D, Jeoung A, Choi ES. Clinical Characteristics and Etiology of Travelers'

Diarrhea among Korean Travelers Visiting South-East Asia. J Korean Med Sci. 2011 Feb; 26(2):196-200.

14. Jeon DY, Lee JC, Song HJ. Molecular Epidemiological Characteristics of Vibrio parahaemolyticus Isolated from Diarrheal Patients in Jeonnam, Korea. J Bacteriol Virol. 2009 Sep; 39(3):229-235.

15. Yeung PS1, Boor KJ. Epidemiology, pathogenesis, and prevention of foodborne Vibrio parahemolyticus infections. Foodborne Pathog Dis.2004 Summer; 1(2)74-88

16. Wang R, Zhong Y, Gu X, Yuan J, Saeed AF, Wang S .The pathogenesis, detection and prevention of Vibrio parahemolyticus. Front Microbiol.2015; 6: 144

17. Distribution of Vibrio Parahaemolyticus in Fishery products, Sold at Garak Wholesale Market and Serological Characteristics of isolated Strains

18. S Nelapati, K Nelapati, B K Chinnam. Vibrio parahaemolyticus- An emerging foodborne pathogen-A Review. Vet. World, 2012, Vol.5 (1): 48-62

19. KCDC. National Infectious Disease Surveillance System, NIDSS.2017:14-17

20. Kim, Ha-Kyu , Lee, Hak-Tae, Kim, Jong-Ho, Lee, Sang-Sun. Analysis of Microbiological Contamination in Ready-to-eat Foods. Journal of Food Hygiene and Safety: volume23.issue4.2008. PP285-290

21. Jang HG, et al .Microbiological quality and risk factors related to sandwiches served in bakeries, cafes, and sandwich bars in South korea. J Food Prot. 2013.

22. Pandemic Vibrio parahemolyticus, Maryland, USA,2012. Emerging Infectious Disease.www.cdc.gov/eid.Vol.20,No.4,April 2014

23. Tan Turk Hsern Malcolm, Wei San Chang, Yuet Ying Loo et al. Simulation of improper food hygiene practices: A quantitative assessment of Vibrio parahaemolyticus distribution. International Jounal of Food Microbiology 284(2018)112-119

24. Mu Ye, Yaoxin Huang, Haiqiang Chen. Inactivation of Vibrio parahaemolyticus and Vibrio vulnificus in oysters by high-hydrostatic pressure and mild heat. Food Microbiology 32(2012)179-18421-2.

25. Yi-Cheng Su, Chngchu Liu. Vibrio parahaemolyticus: A concern of seafood safety. Food Microbiology. Volume 24, Issue 6, September 2007, Pages 549-558

26. Vu TTT, Alter T, Huehn S. Prevalence of Vibrio spp.in Retail Seafood in Berlin , Germany. J Food Prot.2018 Apr:81(4):593-597

감사의 글

지역사회 의료를 성실히 담당하고 역학조사에 적극적으로 도움을 주신 녹색 병원 의료진과 중랑구 보건소 관계자분들께 감사 드립니다
